# Long Term Metabolic Syndrome Induced by a High Fat High Fructose Diet Leads to Minimal Renal Injury in C57BL/6 Mice

**DOI:** 10.1371/journal.pone.0076703

**Published:** 2013-10-03

**Authors:** Romain Dissard, Julie Klein, Cécile Caubet, Benjamin Breuil, Justyna Siwy, Janosch Hoffman, Laurent Sicard, Laure Ducassé, Simon Rascalou, Bruno Payre, Marie Buléon, William Mullen, Harald Mischak, Ivan Tack, Jean-Loup Bascands, Bénédicte Buffin-Meyer, Joost P. Schanstra

**Affiliations:** 1 Institut National de la Santé et de la Recherche Médicale (INSERM), U1048, Institut of Cardiovascular and Metabolic Disease, Toulouse, France; 2 Université Toulouse III Paul-Sabatier, Toulouse, France; 3 Plateau de Protéomique des Liquides Biologiques, Institut of Cardiovascular and Metabolic Disease, Toulouse, France; 4 Mosaiques Diagnostics GmbH, Hannover, Germany; 5 Charite-Universitatsmedizin Berlin, Berlin, Germany; 6 Centre de Microscopie Electronique Appliquée à la Biologie, Toulouse, France; 7 Department of Proteomics and Systems Medicine, BHF Glasgow Cardiovascular Research Centre, Institute of Cardiovascular and Medical Sciences, College of Medical Veterinary and Life Sciences, University of Glasgow, Glasgow, United Kingdom; University of Louisville School of Medicine, United States of America

## Abstract

Metabolic syndrome can induce chronic kidney disease in humans. Genetically engineered mice on a C57BL/6 background are highly used for mechanistic studies. Although it has been shown that metabolic syndrome induces cardiovascular lesions in C57BL/6 mice, in depth renal phenotyping has never been performed. Therefore in this study we characterized renal function and injury in C57BL/6 mice with long-term metabolic syndrome induced by a high fat and fructose diet (HFFD). C57BL/6 mice received an 8 months HFFD diet enriched with fat (45% energy from fat) and drinking water enriched with fructose (30%). Body weight, food/water consumption, energy intake, fat/lean mass ratio, plasma glucose, HDL, LDL, triglycerides and cholesterol levels were monitored. At 3, 6 and 8 months, renal function was determined by inulin clearance and measure of albuminuria. At sacrifice, kidneys and liver were collected. Metabolic syndrome in C57BL/6 mice fed a HFFD was observed as early 4 weeks with development of type 2 diabetes at 8 weeks after initiation of diet. However, detailed analysis of kidney structure and function showed only minimal renal injury after 8 months of HFFD. HFFD induced moderate glomerular hyperfiltration (436,4 µL/min *vs* 289,8 µL/min; p-value=0.0418) together with a 2-fold increase in albuminuria only after 8 months of HFFD. This was accompanied by a 2-fold increase in renal inflammation (p-value=0.0217) but without renal fibrosis or mesangial matrix expansion. In addition, electron microscopy did not show alterations in glomeruli such as basal membrane thickening and foot process effacement. Finally, comparison of the urinary peptidome of these mice with the urinary peptidome from humans with diabetic nephropathy also suggested absence of diabetic nephropathy in this model. This study provides evidence that the HFFD C57BL/6 model is not the optimal model to study the effects of metabolic syndrome on the development of diabetic kidney disease.

## Introduction

The food industry has evolved, providing a supply of increasingly rich diet in terms of fatty acids [1] and fructose [2]. This shift is not without consequences on general health including increase risk of developing obesity, insulin resistance, hepatic steatosis and diabetes [3-10]. Metabolic syndrome is a complex pattern of disorders referring to the joint occurrence of several risk factors including obesity, mixed dyslipidemia and high glucose blood levels [11]. Metabolic syndrome is very common in developed countries and its prevalence is expected to further increase in the near future [12], in parallel with the rapidly increasing prevalence of obesity [13,14]. Metabolic syndrome and its risk factors potentially play a role in the development of chronic kidney diseases (CKD). Indeed, it has been shown that metabolic syndrome is associated with a higher prevalence of microalbuminuria [15-20] and an higher risk of development of CKD [21] and subsequent end stage renal disease (ESRD) in type II diabetic patients [22]. However, detailed mechanistic information on the link between metabolic syndrome and CKD is not available.

**Figure 1:**
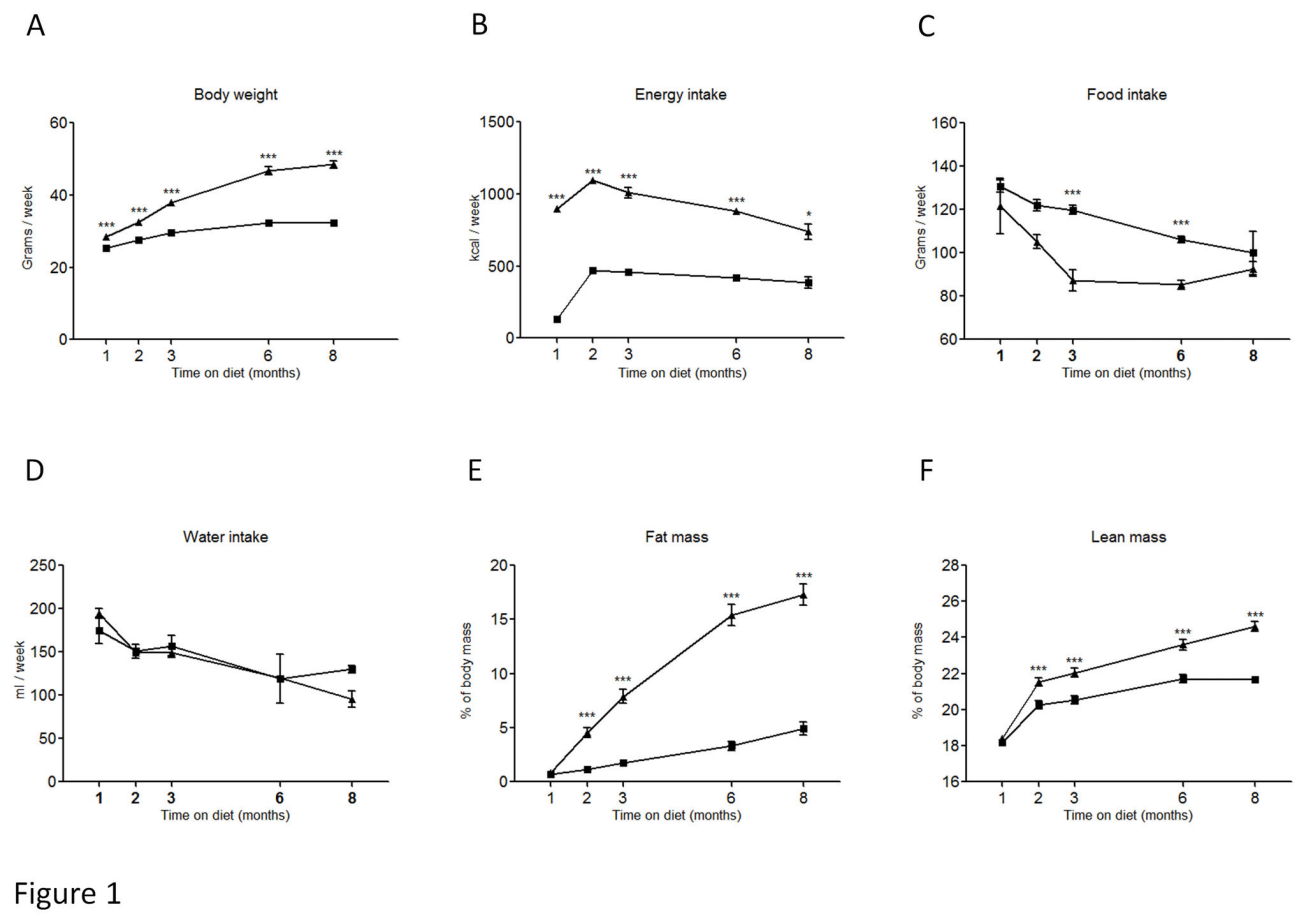
Changes in body weight (A), energy intake (B), food intake (C), water intake (D), fat mass (E) and lean mass (F) in control fed mice (■) and HFFD fed mice (▲). Data are means ± SEM for 36 mice per group at 1, 2 and 3 months, 24 mice per group at 6 months and 12 mice per group at 8 months. *P≤0.05, **P≤0.01, ***P≤0.001.

Hence, to better understand this link, rodent models mimicking as best as possible human metabolic syndrome-induced CKD will be of great help. It has been shown that a 3 or 4 months diet containing respectively 60% or 45% kcal of fat leads to mesangial matrix area expansion in renal glomeruli and to a significant rise in urinary albumin excretion in C57BL/6 mouse [23,24]. Furthermore, the use of fructose alone (between 20% and 40%) in mouse and rat diets has been reported to induce renal tubulointerstitial injury [25,26]. The drawback of these models is that they only mimic certain aspects of metabolic syndrome but not the entire repertoire [27]. In contrast, a combination of high fat and high carbohydrate diet in animals leads to the development of all typical metabolic complications present in human metabolic syndrome such as increased body weight, increased triglycerides and cholesterol plasma concentrations, and abdominal fat deposition [28-31]. The latter is thus probably the most appropriate model to study human metabolic syndrome in animal models [27] and therefore its impact on kidney function. Recent studies by Panchal et al. [32] submitting rats to both a high fat and high fructose diet for 4 months showed cardiovascular remodeling (i.e endothelial dysfunction, inflammation and fibrosis in the heart) in the presence of metabolic syndrome. Unfortunately, effects on the kidney were only minimally described which did not allow concluding on the suitability of this metabolic syndrome model to induce CKD. In addition, the workhorse for genetically engineered mice is the C57BL/6 strain and induction of metabolic syndrome in this strain deficient for specific genes would be of great help to better understand the development of CKD in metabolic syndrome.

Therefore in the current study we describe the in depth characterization of kidney lesions after a long term (8 months) high fat high fructose diet (HFFD) in the C57BL/6 mouse strain. We observed that the kidney is particularly resistant to metabolic syndrome in this strain since only very mild signs of kidney damage were observed after 8 months of HFFD.

**Figure 2:**
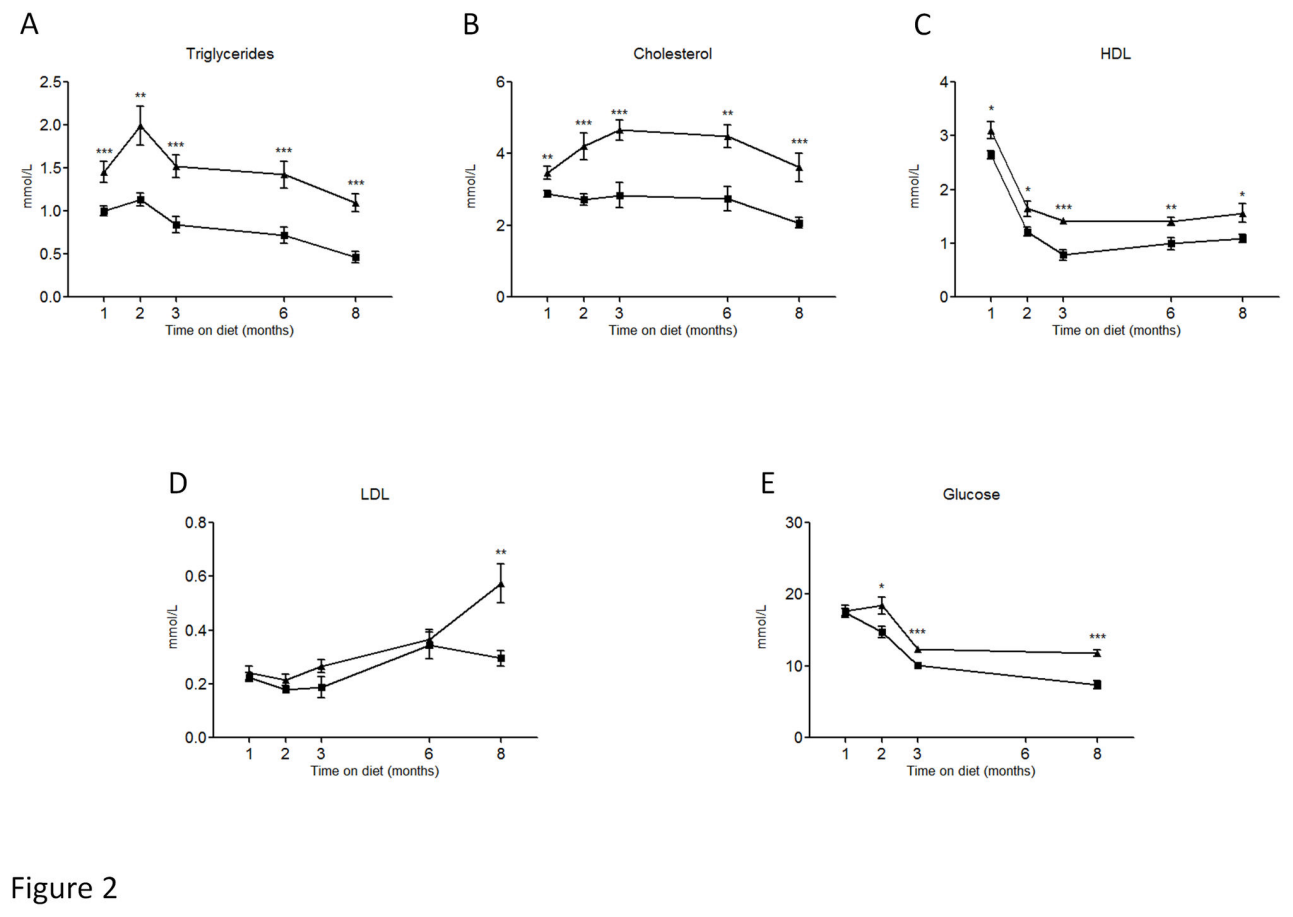
Plasma level of triglycerides (A), cholesterol (B), HDL (C), LDL (D) and glucose (E) in control fed mice (■) and HFFD fed mice (▲) Data are means ± SEM for 12 mice per group. *P≤0.05, **P≤0.01, ***P≤0.001.

## Materials and Methods

### Mice and diets

Seventy-eight adult male C57BL/6 (nomenclature C57BL/6NCrl) mice were purchased at Charles River Laboratory (L’Arbresle, France) at the age of 5 weeks and divided in two equal experimental groups (n=36). Mice were housed 4 per cage and maintained on a 12h light/12h dark cycle in a pathogen-free environment. Mice were fed with either a control diet or a high fat fructose diet (HFFD). The control diet (D12450B; 3,82 kcal per g; Research Diet, USA) contained 19.2% protein, 67.3% carbohydrate and 4.3% fat and was served with regular drinking water; both ad libitum. The HFFD (D12451; 4,73 kcal per g; Research Diet, USA) contained 24% protein, 41% carbohydrate and 24% fat and was served with 30% fructose (F0127 powder diluted in ultrapure water; 1,2 kcal per ml; Sigma, France) enriched drinking water; both ad libitum. All experiments reported were conducted in accordance with the NIH guide for the care and use of laboratory animals and were approved by the animal care and use committee from UMS US006/INSERM, Toulouse, France (protocol 11/1048/12/19).

### Body weight, composition and energy intake

Mice were weighed weekly. The body composition (fat and lean mass) of awake mice was analyzed by nuclear magnetic resonance using an EchoMRI-3in1 apparatus for Live Animals (EchoMRI, USA) [33]. Food intake was estimated as the difference of weight between the offered and the remnant amount of food at 7-days intervals. The food was provided as pressed pellets so the residual spillage was not considered here. Beverage intake was estimated with the same process based this time on the volume. Total energy intake, determined from the energy content in each diet and mass consumed, was calculated on a per-week basis.

### Blood analysis

Prior to blood collection, we performed a 6 hours fasting period. This time point was chosen to avoid a postprandial glycemic peak and to prevent an induced catabolic state in the mice [34,35]. All the blood samples were collected in Microvette CB 300 Plasma/Lithium heparin (Sarstedt, Germany) tubes from the tail vain under local anesthesia (n=12 per group). The samples were centrifuged at 3000 g for 10 minutes at 4°C. The supernatants were separated and plasma concentrations of glucose, cholesterol, HDL, LDL and triglycerides were measured by fluorescence on ABX Pentra 400 apparatus (Horiba medical, France). In order to bypass multiple periods of fasting and blood collection, all the parameters were measured from the same sample.

### Renal function study

After 3, 6 and 8 months of diet, glomerular filtration rate (GFR) was evaluated by measurement of inulin clearance in 9 mice per group as previously described [36]. Briefly mice were anesthetized with an intraperitoneal injection of 150 mg/kg of thiobutabarbital sodium (Inactin, Sigma) and placed on a thermostatically controlled heating table. After a tracheotomy, the left jugular vein was cannulated to infuse Gelofusine and a mixture of NaCl (0.9%), thiopenthal sodium (pentothal, 0.83mg·kg^-1^·min^-1^), inulin (1.8 mg·kg^-1^·min^-1^). The rate of infusions was 0.1 ml/h. The left femoral artery was cannulated to monitor mean arterial blood pressure (MABP) and to obtain blood samples. Urine was obtained in the following way: A 3 mm ventral incision was made opposite to the bladder, below the peritoneum, allowing to expose the bladder. Then a polyethylene catheter (internal diameter 0.58 mm; external diameter 0.96 mm) was introduced in the bladder lumen by a 1 mm incision. The bladder was then tied around the catheter using surgical suture. The residual urine is removed from the bladder and a 30 min period of recovery is observed. During GFR evaluation, a 500 µl tube is disposed at the free extremity of the catheter to collect urine during 60 minutes. At the end of surgery, a bolus of inulin (inulin: 100 mg/kg) was perfused, and mice were allowed to recover for 30 min. Renal function was evaluated for a 60-min clearance period. Inulin levels in plasma and urine samples were determined by enzymatic quantification of released fructose units (Enzytec D-Glucose/Fructose kit, r-biopharm^®^) on a Pentra 400 analyzer (Horiba Medical^®^) after a one hour 37°C incubation of the samples with inulinase (Sigma^®^). The GFR is equal to the inulin clearance (GFR = ([urinary inulin] x urine flow) / [inulin]).

### Albuminuria

Mice were placed in metabolic cages in order to collect 24-h urine. Urinary albumin excretion was determined with a mouse antigen specific ELISA (Mouse Albumin Quantitation Kit, Bethyl Laboratories®, USA). Creatinine measurements were determined by an enzymatic colorimetric assay using an ID-MS traceable calibration (Creatinine Enzymatic, Beckman Coulter) on the Pentra 400 analyzer (Horiba Medical^®^). Albumin excretions were related to urine creatinine concentrations in order to take into account the differences in urinary flows.

**Figure 3:**
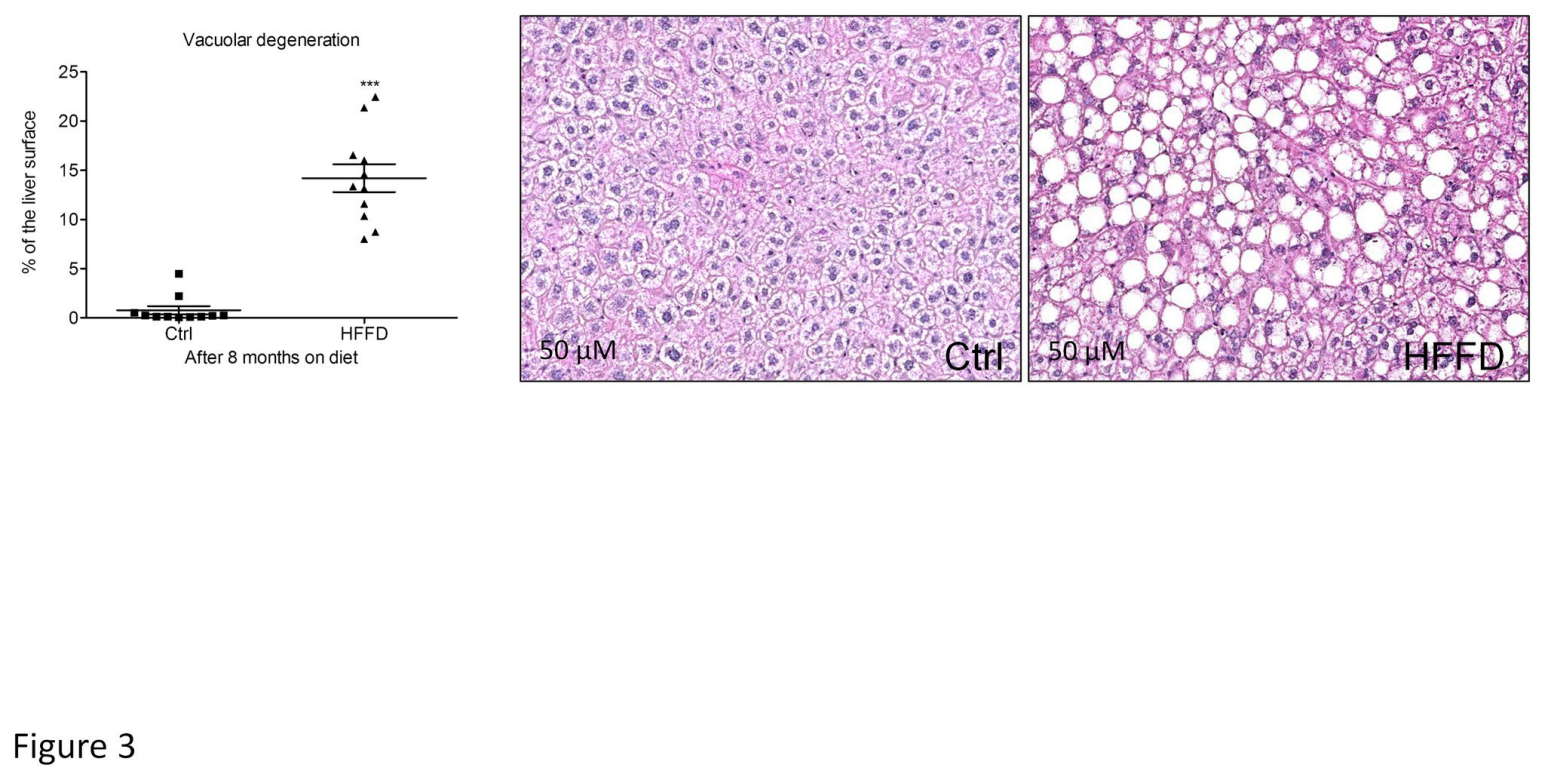
Marked vacuolar degeneration indicating fat accumulation in the liver of HFFD fed mice (■) compared to control fed mice (▲) Hematoxylin/eosin staining. Data are means ± SEM for 12 mice per group. ***P≤0.0001.

### Organ collection

Kidneys and liver were collected from each mouse included in the renal function study. For brightfield microscopy, tissues were fixed in Carnoy’s solution for 24 h. For electron microscopy, tissues were fixed and stored with 2% glutaraldehyde in Sorensen buffer for 1 hour.

### Histological analysis and immunochemistry

Four-micrometer paraffin-embedded sections were cut. Renal and liver sections were subjected to histological analysis, using hematoxylin-eosin and periodic acid-Schiff staining. For immunohistochemistry experiments, renal sections were first dewaxed in toluene and rehydrated through a series of graded ethanol washes before endogenous peroxidase blockage (S2001, DakoCytomation, Trappes, France). Specific primary antibodies were incubated (1h at room temperature) for the detection of collagen type III (1/500; Tebu-Bio, Le Perray-en-Yvelines, France) and F4/80 positive cells (1/250; RM2900; Caltag Laboratories Inc., Burlingame, CA, USA). Slides were further incubated with peroxidase-conjugated secondary antibodies during 30 min. Immunological complexes were visualized by the addition of the DAB substrate during 10 min (K4010, DakoCytomation Envision HRP system). Sections were counterstained with hematoxylin and mounted. Negative controls for the immunohistochemical procedures included substitution of the primary antibody with nonimmune sera. Sections were scanned using a Nanozoomer 2.0 RS (Hamamatsu Photonics SARL, Massy, France) and treated with the Morpho-expert image-analysis software (version 1.00, Explora Nova, La Rochelle, France) for morphometric analyses.

**Figure 4:**
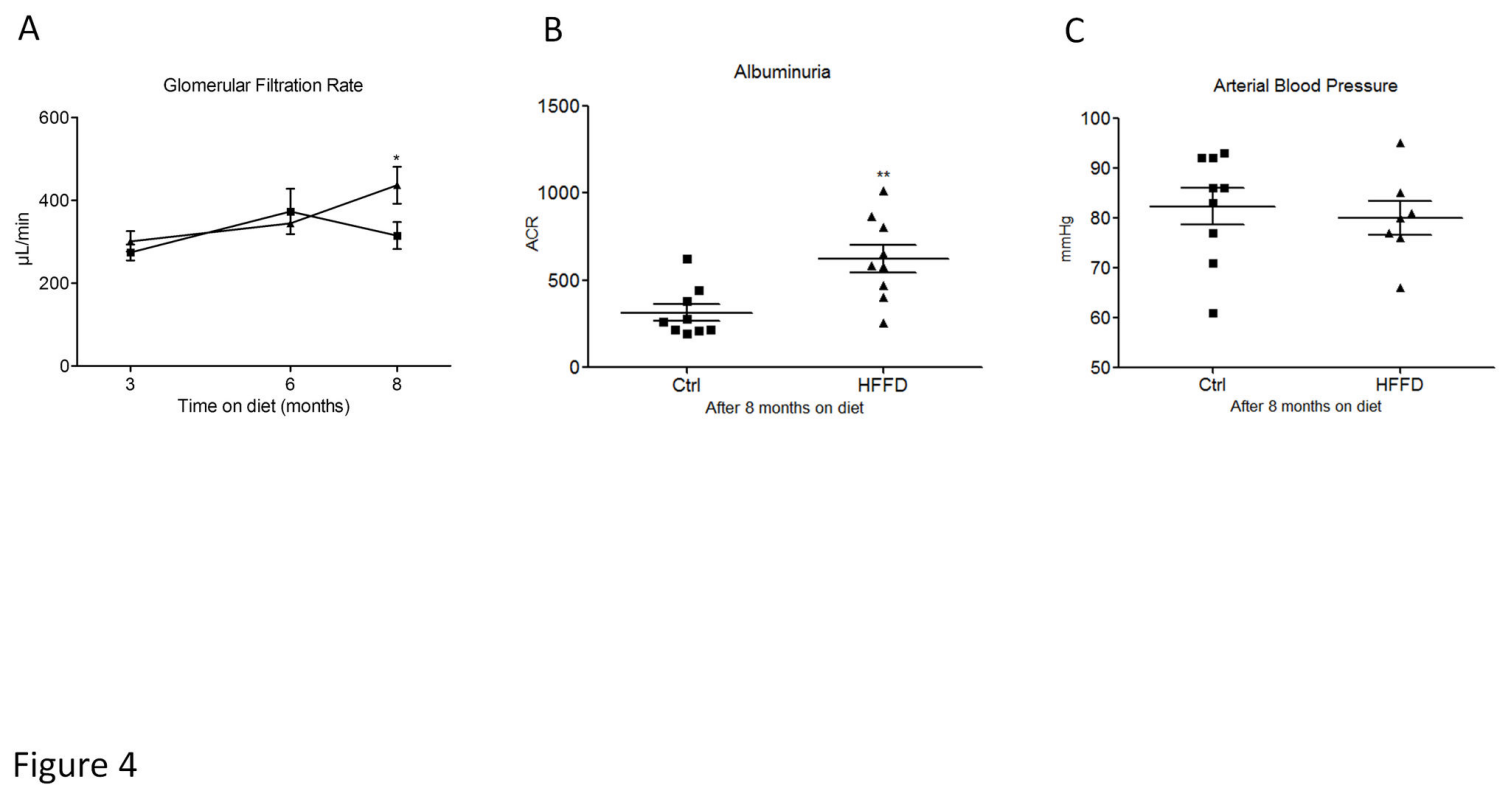
Glomerular hyperfiltration (A), urinary albumin to creatinine ratio (ACR) (B) and arterial blood pressure (C) in HFFD fed mice (■) compared to control fed mice (▲) Data are means ± SEM for 9 mice per group; *p=0.0429; **p=0.004.

### Transmission electron microscopy

Samples were fixed with 2% glutaraldehyde in Sorensen phosphate buffer (0.1 M, pH 7.4) for 1 hour, washed with the Sorensen phosphate buffer for 12 hours and post fixed with 1% OsO_4_ in Sorensen phosphate buffer for 1 hour. Samples were dehydrated with increasing ethanol concentration (30%, 50%, 70%, 95%) until a 100% ethanol solution was reached and then subsequently stored for 30 min in a propylene oxide solution. Next, samples were embedded in epoxy resin (Epon 812). After 24 h of polymerization at 60°C, ultrathin sections (70 nm) were mounted on 100 mesh collodion-coated copper grids and poststained with 3% uranyl acetate in 50% ethanol and with 8.5% lead citrate before being examined on a HT7700 Hitachi electron microscope at an accelerating voltage of 80 KV. A series of 10 photographs were taken at x12000 magnification. For assessment of basement membrane thickness, a grid (grid size = 30cm by 30 cm with a 0.5 cm step) was projected on each photograph and basement membrane thickness was measured at points where it intersected with the grid [37]. For assessment of foot process effacement, the number of podocytic foot processes was manually counted and expressed as the number of foot processes per µm of glomerular basement membrane length [38].

**Figure 5:**
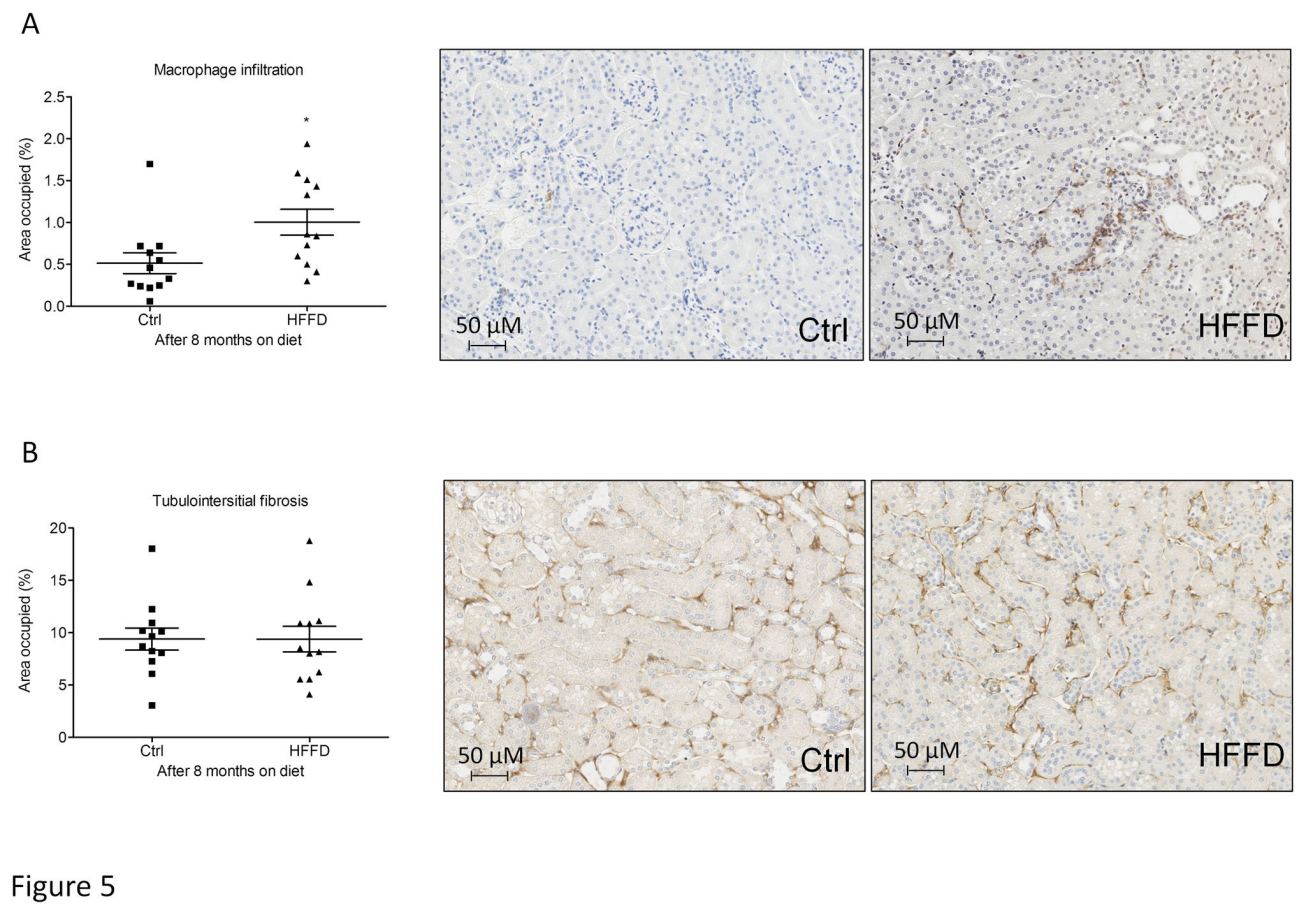
Macrophage infiltration (A) and collagen accumulation (B) in HFFD fed mice (■) compared to control fed mice (▲) Data are means ± SEM for 12 mice per group; *p=0.0217. Pictures for macrophage staining display representative areas of kidneys from control or HFFD fed mice. Magnification x20. Pictures for collagen staining display representative tubulointerstitium of kidneys from control or HFFD fed mice. Magnification x20.

### Urine sample preparation for CE-MS analysis

For the analysis of the urinary peptidome, urine samples from all mice of each group were collected during 12 hours using a metabolic cage and frozen at -80°C until CE-MS analysis. Urine for peptidome analysis was obtained 2 days prior to inulin based GFR measurements. Immediately before preparation, mouse urine aliquots were thawed and 150 µl was mixed with 150 µl of a buffer 2 M urea, 10 mM NH4OH containing 0.02% SDS. Subsequently, samples were ultrafiltered using a Centristat 20 kDa cut-off centrifugal filter device (Satorius,Gottingen, Germany) to eliminate high molecular weight compounds. The obtained filtrate was desalted using a NAP5 gel filtration column (GE Healthcare Bio Sciences, Uppsala, Sweden) to remove urea and electrolytes. The sample was lyophilized in a Christ Speed-Vac RVC 2 18/Alpha 1 2 (Christ, Osterode am Harz, Germany) and stored at 4°C until use. Finally, the samples were re-suspended in 200 µL HPLCgrade H2O and injected into CE-MS with 2 psi for 99 sec, resulting in injection volumes of 280 nL. Capillary electrophoresis coupled to mass spectrometry (CE-MS) analysis was performed as described using a P/ACE MDQ capillary electrophoresis system (Beckman Coulter, Fullerton, USA) on-line coupled to a MicroQTOF MS (Bruker) [39]. The ESI sprayer (Agilent Technologies, Palo Alto, CA, USA) was grounded, and the ion spray interface potential was set between −4.0 and −4.5 kV. Data and MS acquisition methods were automatically controlled by the CE via contact-close-relays. Spectra were accumulated every 3 s, over a range of m/z 350 to 3000. Details on accuracy, precision, selectivity, sensitivity, reproducibility, and stability of the CE-MS method have been provided previously [40,41]

### Peptide sequencing

For sequencing, processed urine samples were also separated on a Dionex Ultimate 3000 RSLS nano flow system (Dionex, Camberly UK). A 5 ml sample was loaded onto a Dionex 5 mm C18 nano trap column at a flow rate of 5 ml/min. Elution was performed on an Acclaim PepMap 75 mm C18 nano column over 100 min. The sample was ionised in positive ion mode using a Proxeon nano spray ESI source (Thermo, Fisher Hemel UK) and analysed in an Orbitrap Velos FTMS (Thermo Finnigan, Bremen, Germany). The MS was operated in data-dependent mode to switch between MS and MS/MS acquisition and parent ions were fragmented by (high-) energy collision-induced dissociation and also electron transfer dissociation. Data files were searched against *Mus musculus* entries in the Swiss-Prot database without any enzyme specificity using Open Mass Spectrometry Search Algorithm (OMSSA, http://pubchem.ncbi.nlm.nih.gov/omssa) with an e-value cut-off of 0.1. No fixed modification and oxidation of methionine as variable modifications were selected. Mass error windows of 10 ppm for MS and 0.05 Da (HCD) or 0.5 Da (CID, ETD) for MS/MS were allowed. For further validation of obtained peptide identifications, the strict correlation between peptide charge at pH 2 and CE-migration time was utilized to minimize false-positive identification rates [42]. Calculated CE-migration time of the sequence candidate based on its peptide sequence (number of basic amino acids) was compared to the experimental migration time. Peptides were accepted only if they had a mass deviation below ± 80 ppm and a CE-migration time deviations below ± 2 min.

### Data processing

Mass spectral ion peaks representing identical molecules at different charge states were deconvoluted into single masses using MosaiquesVisu software [43]. Only those signals with z >1 that were observed in a minimum of 2 consecutive spectra with signal-to-noise ratios >4 were included. The software employs a probabilistic clustering algorithm and uses both isotopic distribution as well as conjugated masses for charge-state determination of peptides/proteins. The resulting peak list characterizes each polypeptide by its molecular mass, CE-migration time, and ion signal intensity (amplitude) value. To minimize effects of biological and analytical variability between the different lots, a normalization of retention time, signal intensity and mass was performed as described [44]. All detected peptides were deposited, matched, and annotated in a Microsoft SQL database, allowing further analysis and comparison of multiple samples [45]. Peptides were considered identical within different samples when mass deviation was lower than 50 ppm for small peptides or 75 ppm for larger peptides and proteins. Due to analyte diffusion, CE peak widths increase with CE migration time. In the data clustering process this effect was compensated by linearly increasing cluster widths over the entire measurement from 2 to 5%.

**Figure 6:**
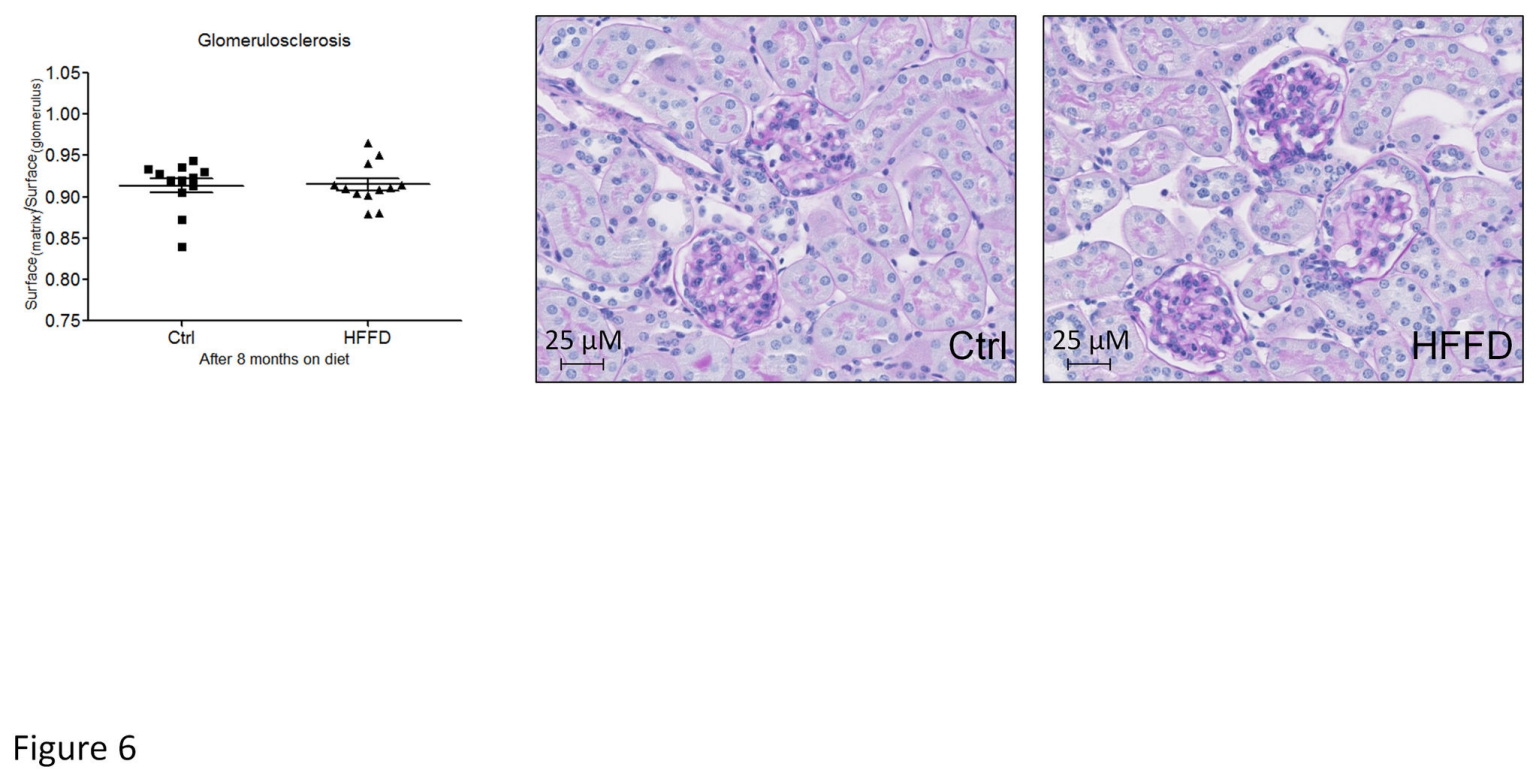
Absence of mesangial matrix expansion in HFFD fed mice (■) compared to control fed mice (▲) Mesangial matrix expansion is evaluated by the ratio of the matrix surface to the total surface of the glomerulus after Periodic Acid Staining. Data are means ± SEM for 12 mice per group; *ns*. Pictures display representative glomeruli of kidneys from control or HFFD fed mice. Magnification x40.

### Statistical analysis

All data are presented as means ± SEM. Statistical analysis was performed using GraphPad software (www.graphpad.com). Analysis of the two groups (control *vs.* high fat fructose diet) was assessed by a two-tailed unpaired t-test. Statistical significance was reached at a *p-value* below 0.05.

## Results

### HFFD diet induces metabolic syndrome and T2D in mice

Five weeks old male C57BL/6 mice were separated into two groups (n=36 mice/group). The control group received a standard control diet and tap water. The other group (HFFD) received a high fat diet (45% of fat) and 30% fructose in tap water. In Table 1, we summarized the parameters to determine instauration of metabolic syndrome in HFFD fed C57BL/6 mice. The different parameters are ordered based on first appearance of significance in time. Mice receiving HFFD displayed a significantly higher energy intake starting at week 1. Body weight of the HFFD group was increased starting at week 3 while the food intake in this group started to decrease. After 4 weeks on HFFD, we observed that plasma triglycerides, total cholesterol, and HDL were significantly elevated. The difference observed for weight between HFFD and control mice was confirmed by a significant gain of fat and lean mass in HFFD mice at week 8. At the same time, the HFFD mice started to be hyperglycemic compared with controls. At week 16, plasma LDL levels were significantly higher in the HFFD mice. The figures 1 and 2 show the evolution of these parameters throughout the eight months of study. No difference was observed in the water intake. Histological analysis of the liver performed at 8 months revealed a marked vacuolar degeneration, indicating fat accumulation in the liver (Figure 3). This observation is consistent with the development of non alcoholic fatty liver (NAFLD) due to the excess of fructose intake [46]. Collectively these data strongly suggest the presence of metabolic syndrome as early as 4 weeks and of associated type 2 diabetes (T2D) as early as 8 weeks after initiation of a HFFD in C57BL/6 mice.

**Figure 7:**
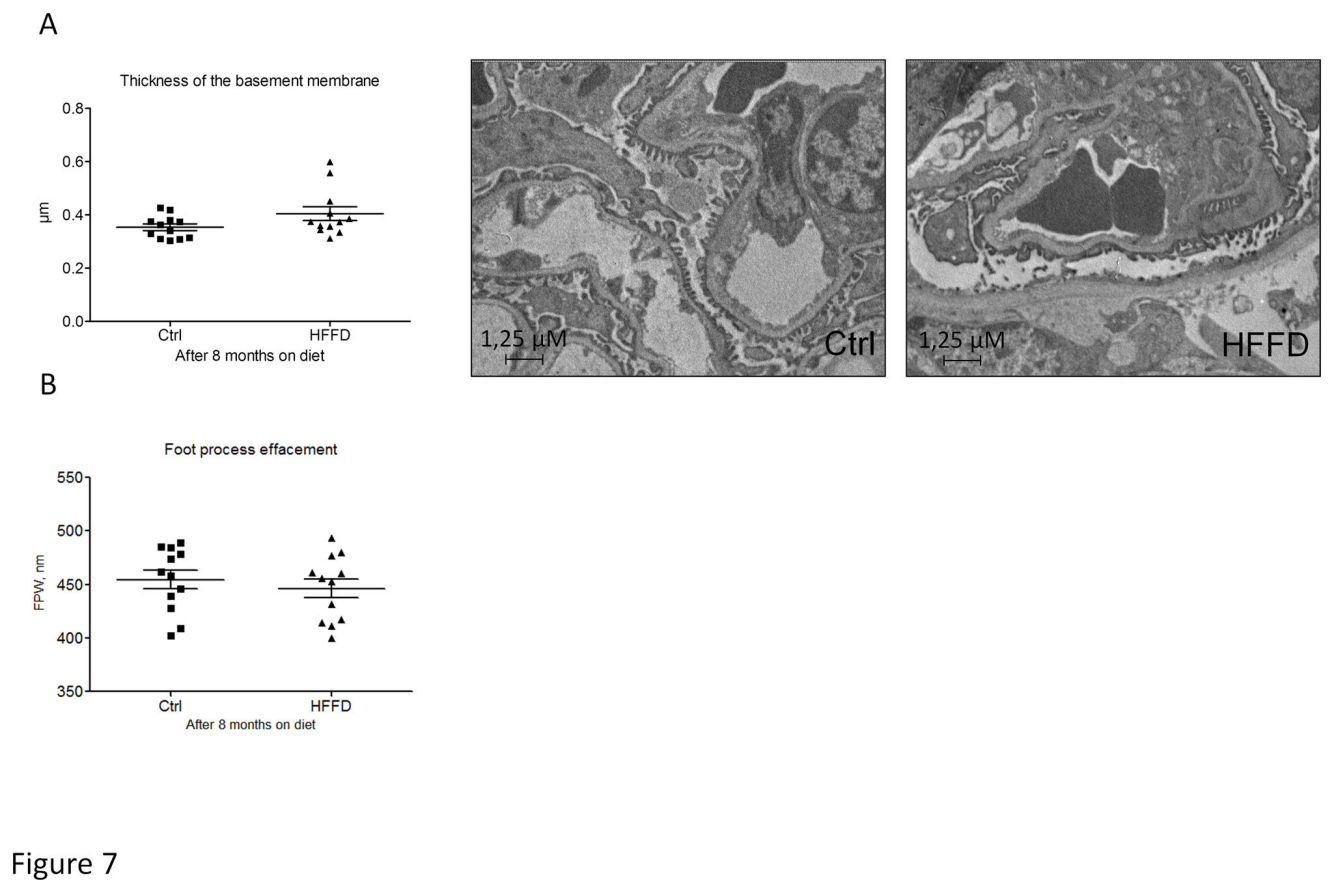
The measurement of glomerular capillary basement membrane thickness by electron microscopy showed a tendency to increase in the HFFD group at 8 months (A). HFFD fed mice (■) compared to control fed mice (▲) and data represent means ± SEM for 12 mice per group; *ns*. The assessment of foot process effacement, expressed as the mean width of the podocytes foot process (FPW), did not showed a significant difference between the HFFD fed mice (5) compared to control fed mice (<) (B). Data represent means ± SEM for 12 mice per group; *ns*.

**Table 1:** Parameters to determine instauration of metabolic syndrome in HFFD fed C57BL/6 mice.

	**Onset of significance**	**Control group values**	**HFFD group values**	**p-value**	**Test frequency**
**Energy intake (kcal)**	1 week	456.2 ± 12.3	1398 ± 129.2	< 0.0001	weekly
**Body weight (grams)**	3 weeks	24.88 ± 0.19	26.79 ± 0.23	< 0.0001	weekly
**Food intake (grams)**	3 weeks	160.0 ± 8.17	96.33 ± 9.26	0.0004	weekly
**Triglycerides (mmol/L)**	4 weeks	1.00 ± 0.07	1.45 ± 0.12	0.0023	monthly
**Cholesterol (mmol/L)**	4 weeks	2.89 ± 0.09	3.46 ± 0.18	0.0083	monthly
**HDL (mmol/L)**	4 weeks	2.64 ± 0.08	3.10 ± 0.16	0.0148	monthly
**Fat mass (% of body mass)**	8 weeks	1.16 ± 0.11	4.55 ± 0.45	< 0.0001	monthly
**Lean mass (% of body mass)**	8 weeks	20.26 ± 0.24	21.53 ± 0.22	0.0003	monthly
**Glucose (mmol/L)**	8 weeks	14.77 ± 0.79	18.41 ± 1.21	0.0164	monthly
**LDL (mmol/L)**	16 weeks	0.14± 0.01	0.20 ± 0.02	0.0071	monthly

The different parameters are ordered based on first appearance of significance in time. The frequency of analysis for each parameter is displayed in the last column. n=36 / group.

**Figure 8:**
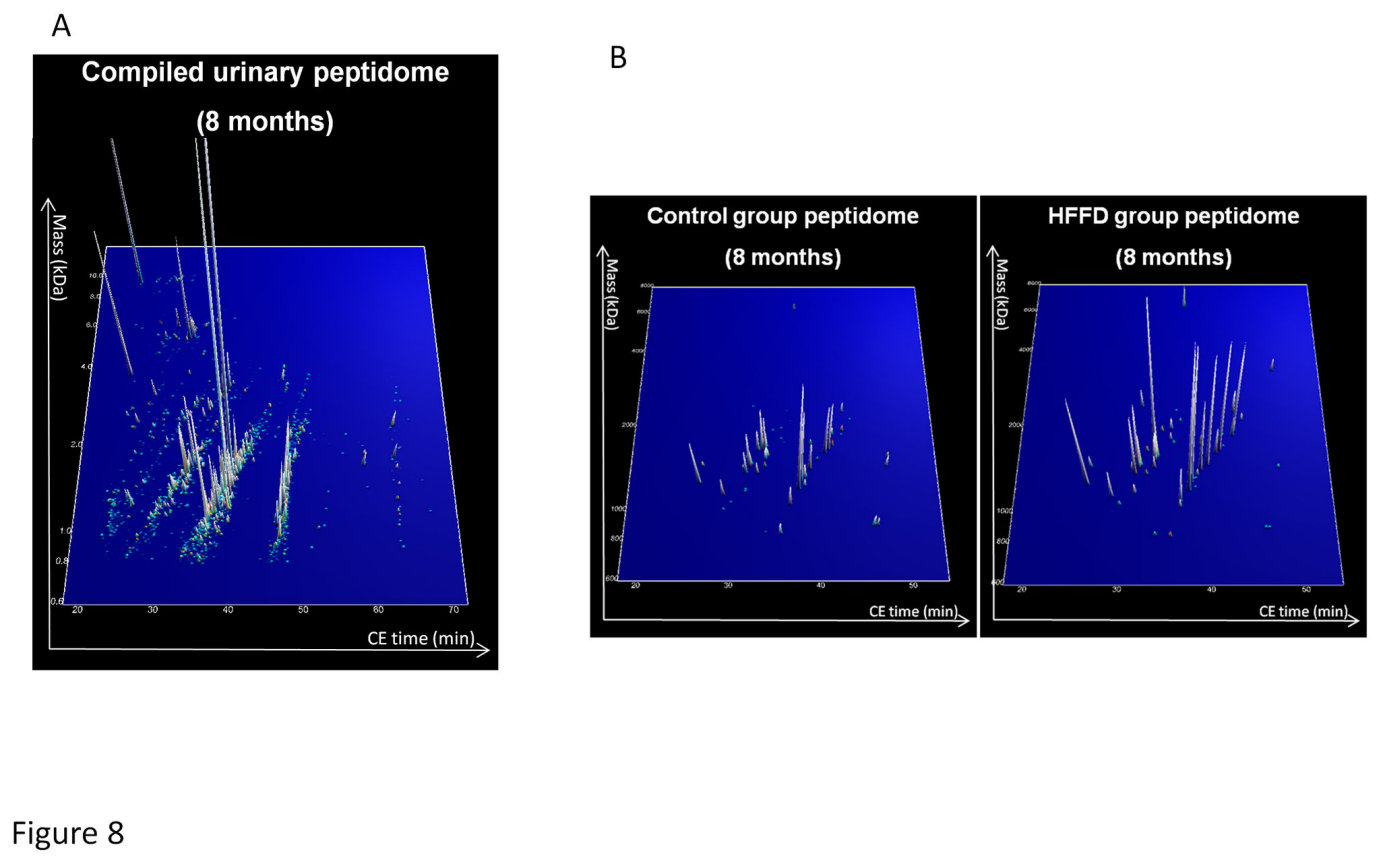
Comparison of the urinary peptidome content between control (n=10) and HFFD (n=11) at 8 months of age mice. (A) A contour plot representing the compiled urinary peptidome of all HFFD mice showing 2687 urinary peptides. Each blue to white colored dot or peak represents a urinary peptide identified by a specific mass (kDa) and migration time on the CE (min). When a peptide is detected but of low abundance it is represented by a blue dot. Increasing abundance of peptide is represented by a change of color towards white. At the same time the abundance is given by the height of the peak. Therefore a dot equals low abundance and a peak equals higher abundance, with the height of the peak being the relative abundance compared to the other peptides. (B) Representation of the 62 peptides which differ in urinary abundance between control and HFFD fed mice. In panel B, the scale was amplified 2-fold to improve the representation of the peptides.

### HFFD diet induces glomerular hyperfiltration and microalbuminuria only in late stage metabolic syndrome

Next we studied the effect of metabolic syndrome and T2D on kidney function. The glomerular filtration rate (GFR) was determined based on inulin clearance after 3, 6 and 8 months of HFFD. GFR was not significantly different between the control and the HFFD group at the early time points (3 and 6 months), but HFFD mice displayed low, but significant, hyperfiltration after 8 months of diet (Figure 4A). This hyperfiltration was accompanied by a 2-fold increase in albuminuria (Figure 4B), but without change in the blood pressure (Figure 4C). This assessment suggests late and mild kidney dysfunction in metabolic syndrome in C57BL/6 mice.

**Figure 9:**
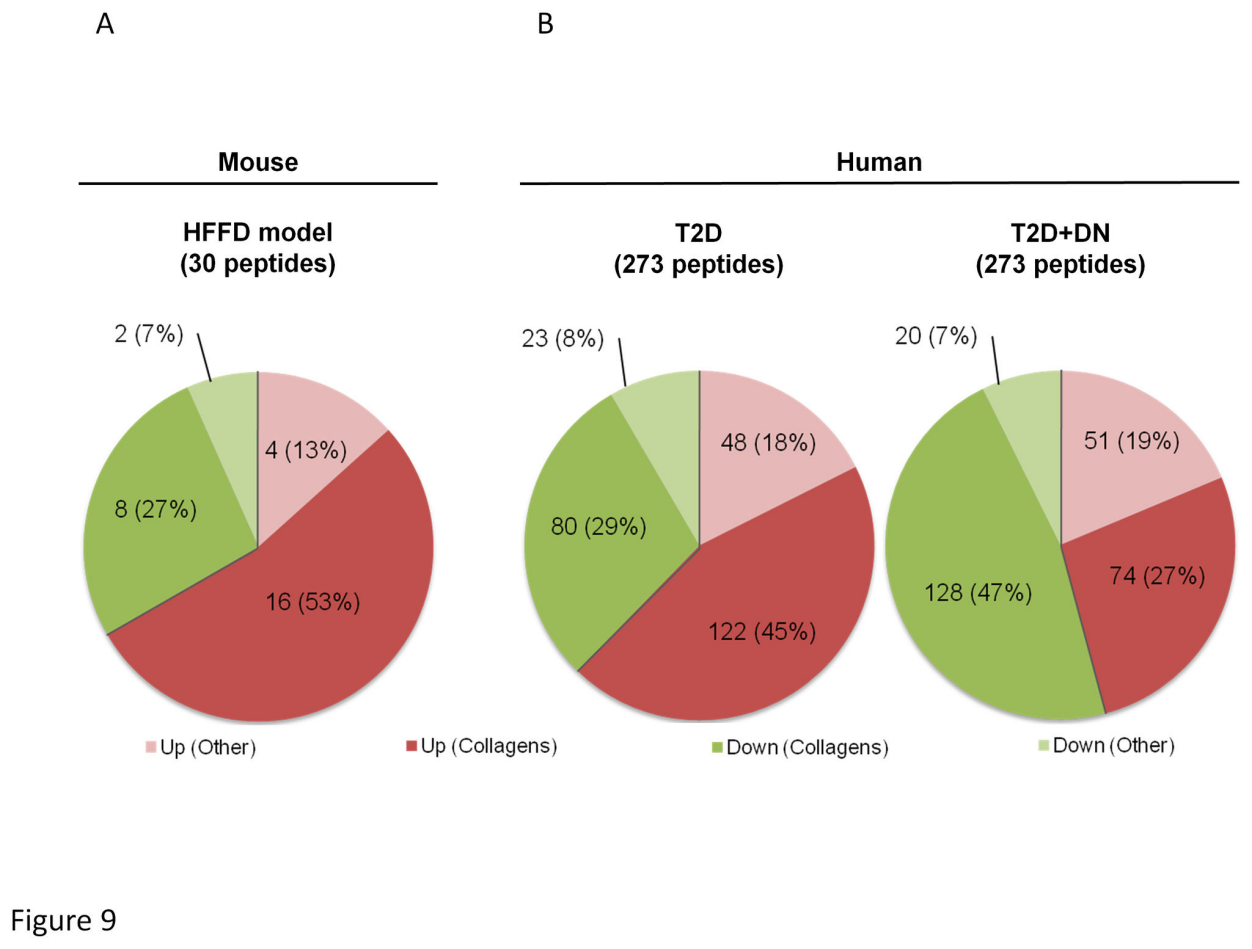
Comparison of the regulation of urinary peptide fragments in the HFFD mouse model (a) with human with type 2 diabetes (T2D) with or without diabetic nephropathy (9B).

### HFFD induces only minor histological modifications in the kidney

Since we only observed mild functional changes after 8 months of HFFD, we studied kidney histological modifications at this time point. Macrophage infiltration was quantified by immunohistological staining for F4/80 in the tubulointerstitium. We observed a 2-fold increased F4/80 staining in kidneys of HFFD mice compared to the control group (Figure 5A). Tubulointerstitial collagen accumulation was also studied by immunostaining for collagen III (Figure 5B). Eight months of HFFD was without effect on tubulointerstitial collagen III deposition. Moreover Periodic Acid staining did not show mesangial matrix expansion (Figure 6). Glomerular basement membrane (GBM) thickening and foot process effacement are early events in diabetic nephropathy [47]. We studied the thickness of the GBM and assessed the foot process effacement using electron microscopy. This analysis showed a non-significant difference (0.353±0.009 vs 0.375±0.0106 µM) towards increased GBM thickness and no foot process effacement in HFFD mice (Figure 7). These data suggest absence of advanced diabetic nephropathy lesions but inflammation of the kidney tissue after 8 months of HFFD.

### Comparison of the HFFD mouse urinary peptidome with the human urinary peptidome of T2D patients suggests absence of T2Ddiabetic nephropathy

The results obtained so far suggest that 8 months of HFFD was not associated with significant renal lesions in C57BL/6 mice. In previous studies in humans, analysis of the urinary proteome allowed the identification of 273 peptides associated to CKD [40]. These peptides were also significantly modified in T2D patients with diabetic nephropathy [48] [49]. Similarly, we have studied the HFFD-induced changes in the mouse urinary peptidome. The analysis of the urinary peptidome of control (n=10) and mice fed 8 months of HFFD (n=11) led to the detection of a total of 2591 urinary peptides for control mice and 2687 peptides for HFFD mice (Figure 8A, shown for HFFD mice). When comparing the urinary peptide content of control and HFFD mice we observed 62 peptides with different urinary abundance (Figure 8B). To date, sequence analysis identified 30 peptides of these 62 (Table S1). Around 80% (24 out of 30) of these peptides were found to be fragments of collagen with 27% being down-regulated and 53% up-regulated (Figure 9A). For comparison with the human situation we next studied the regulation of the 273 peptides associated to CKD in healthy controls (n=33, data obtained from [40]), in patients with T2D without diabetic nephropathy (albuminuria<30mg/L, n=75) and in patients with T2D with diabetic nephropathy (albuminuria>300mg/L, n=47) that were previously studied by Molin [50]. In the T2D patients without diabetic nephropathy, the peptidome profile was very similar to HFFD mice, with 29% of collagen fragments down-regulated and 45% up-regulated compared to healthy controls (Figure 9B). However, the peptidome of HFFD mice was different from T2D patients with diabetic nephropathy as in these patients, collagen fragments were mostly down-regulated (47% down- and 27% up-regulated, Figure 9B).

## Discussion

The purpose of our study was to investigate renal lesions after long term (8 months) of high fat high fructose diet-induced metabolic syndrome in the highly used and studied C57BL/6 mouse strain. We confirmed that consumption of a 45% kcal fat with 30% fructose water diet by wild type C57BL/6 led to the rapid installation of metabolic syndrome (4 weeks) and T2D (8 weeks). We next studied in detail the renal phenotype and compared our results to the specifications of the Diabetic Complications Consortium (DiaComp; www.diacomp.org). The DiaComp research criteria for validating a progressive mouse model of diabetic nephropathy implies a decline greater than 50% in GFR over the lifetime of the animal, an increase greater than 10 fold in albuminuria, advanced mesangial matrix expansion, tubulointerstitial fibrosis and glomerular basement membrane thickening superior by 50% over baseline. During our study, we observed that mice with metabolic syndrome develop minimal renal injury exemplified by low level albuminuria (2 fold higher than the control group) or mild hyperfiltration (1.5 fold higher than the control group) after 8 months of HFFD. In addition, we observed neither mesangial matrix expansion nor fibrosis or significant GBM thickening, but only some minor podocyte effacement. Clearly these characteristics are far from the DiaComp recommendations.

Analysis of human urine proteomic profile allows early diagnosis of kidney diseases such as CKD [51] or diabetic nephropathy [48] and increasingly, proteomics are involved in the clinical diagnosis [52,53]. Here, we compared the urinary peptide content of control mice to HFFD mice and identified 62 differentially excreted peptides. We obtained sequence information for 30 of these 62 peptides and we compared those to the human urinary peptide markers of diabetic nephropathy [48]. In both human and mice, most of the urinary peptides were collagen fragments suggesting similarities between the human and C57BL/6 mouse urinary peptidome. Interestingly, the observed “up and down” collagen peptide profile in HFFD mice was very similar to that of T2D patients without diabetic nephropathy but clearly different from that of T2D patients with diabetic nephropathy. This result suggests absence of diabetic nephropathy in HFFD mice, thereby confirming the functional and histological observations. In addition, this inversion of the direction regulation of urinary collagen fragments in diabetic nephropathy in humans, i.e. a significant decrease in urinary collagen fragments, is thought to be related to increased collagen deposition in the diabetic kidney [54] and correlates to the absence of tubulointerstitial fibrosis in mice receiving 8 months of HFFD. Overall, these data based on urinary peptidome analysis further suggest that the HFFD diet does not ‘‘model’’ chronic kidney disease in T2D patients.

Five stages in the development of renal changes in human diabetes have been described [55], but an official description of staging of diabetic nephropathy in mice is not available. Based on our results, 8 months of HFFD diet in C57BL/6 mice corresponds best to human stage 1 renal changes in diabetes (hyperfiltration, absence of thickened GBM and slightly elevated urinary albumin). Together with our other observations of absence of diabetic nephropathy it is clear that the C57BL/6 strain is not the most adequate strain for studies of renal lesions in HFFD induced T2D.

The influence of the genetic background on the disease phenotype has been described in mice [56]. A number of studies already described the impact of the mouse strain on the responsiveness to treatment. C57BL/6 mice are for example relatively resistant to STZ-induced type 1 diabetes compared to DBA/2J and KK/H1J mice [57,58]. The genetic background can also have significant impact on the responsiveness to a specific diet. C57BL/6 mice fed with a high fat high carbohydrate diet displayed increased body weight, had more overall fat, and particularly increased adipose tissue in comparison with A/J mice, although developing similar levels of hyperglycemia, hyperinsulinemia, and hypercholesterolemia [59,60]. The genetic background has also been reported to influence the outcome of CKD or glomerulosclerosis. In reversible unilateral ureteral obstruction [61], the development of CKD was observed in C57BL/6 mice but not in BALB/c. On the contrary, in a 5/6 nephrectomy model, C57BL/6 mice presented only mild glomerulosclerosis in contrast to both 129/Sv and Swiss mice [62]. Hence one of the possibilities to improve on the development of kidney disease in HFFD is testing different mouse strains. However deviation from the C57BL/6 strain rules out the use of the wealth of genetically engineered mice on this genetic background.

Our choice to use a high fat high fructose diet was driven by its high similarity with the actual human western diet and by the panel of symptoms it causes (eg hyperinsulinemia, insulin resistance, impaired glucose tolerance, increase abdominal fat deposition, hepatic steatosis and inflammation). The hypothesis to study the renal complications in this established model of metabolic syndrome was supported by impaired renal function and the renal lesions observed in studies using high fat or high fructose separately in rodents [23-26]. Surprisingly, the combination of these two diets, although clearly inducing metabolic syndrome, did not lead to the expected kidney damage. A possible explanation of the absence of effect on renal lesions of the HFFD is, when compared to a high fat diet only [23], that the quantity of the daily ingested fat in our experiments is lower (0.52 g of fat/day for HFFD vs 0.62g of fat/day for high fat diet). The additional calories provided by the fructose beverage are compensated by decrease in food intake [63]. This minor daily difference in fat intake over a long period (i.e. 8 months) potentially explains the observed difference in renal lesions. Another possible explanation could be modified water intake due to the presence of fructose. Indeed, as described by Bouby et al. [64], glucose in drinking water can increase water intake, slowing down the progression of renal injury in rats. However, we observed that the high concentration of fructose in the drinking water did not lead to a higher water intake.

Thus the current challenge is to find the balance between diets that lead quickly to advanced renal disease (but that do not mimic the situation from human nutritional perspective) and diets that need a longer time of exposure to induce the same stage of disease (but imitating closely the human nutritional input). An option to accelerate renal lesions in C57BL6 mice could be to superimpose high fat high fructose diets to strains predisposed to obesity such as the Lep^ob^, Lepr^db^, KK.Cg-A^y^ strain. Indeed, Zhang et al. [65] combined a strain predisposed to obesity (db/db) and a high fat diet and showed that a high fat diet exacerbates nephropathy and causes early renal failure. However instauration of renal failure was fast (4 weeks) and does not correspond to chronic disease observed in humans. Once again, a balance needs to be found between models that quickly lead to severe renal disease and models that allow studies of development of chronic disease. Moreover, most of the knockout animals are on the C57BL6 background. The use of these genetic models of T2D could necessitate crossing them with knockout animals in futures studies, but genetic T2D model are associated with issues such as reduced fertility [66] and many backcrosses (around 10) to reach a unique (99.9%) new genetic background will be necessary. Increasing the quantity of fat or fructose could be also considered to accelerate the renal lesions but a review of existing literature has highlighted their serious consequences. Using of a 60% fat diet instead of the 45% on this study would quickly induced obesity on a much shorter time but should be considered extreme from a nutritional perspective. In the same way, some studies use fructose concentrations up to 55%. If we assumed that the fatty liver, a symptom of metabolic syndrome, is earlier observed in these mice we cannot overshadow the increased risk of severe NAFLD and hepatic necroinflammatory [67]

An ultimate option to accelerate renal lesions in C57BL/6 mice subjected to a HFFD is to remove one of the kidneys before initiation of the diet. Indeed, it has been shown that uninephrectomy leads to exacerbation of the lesions of diabetic nephropathy in db/db obese mice [68]. In addition, chronic ingestion of fructose worsens the decrease of renal function, proteinuria, and glomerulosclerosis induced by subtotal nephrectomy in the rat [23-26].

In conclusion, the present study demonstrates that the HFFD is an efficient animal model of metabolic syndrome in C57BL/6 mice but suggests that the kidney is particularly resistant in this strain because only very early signs of kidney damage are observed. Potential solutions to exploit this model for advanced renal lesions in C57BL/6 mice include prolongation of the diet, which is feasible but clearly time consuming or, accelerate disease progression by coupling a HFFD with unilateral nephrectomy.

## Supporting Information

Table S1 **List of all significant regulated peptides at control vs. HFFD.** Given are Identification (internal database of Mosaiques diagnostics), mass in Dalton, Capillary electrophoresis migration time in minutes, mean amplitude at controls, mean amplitude at HFFD, regulation factor at disease, AUC (Area under Curve, Receiver operating statistics), p-value adjusted (Benjamini and Hochberg), amino acid sequence (modified amino acids: p=hydroxyproline; k=hydroxylysine; m=oxidized methionine).
(XLS)
